# A new species of *Dicranocentrus* Schött from Hainan (China) with a key to the Chinese species of the genus (Collembola, Entomobryidae)

**DOI:** 10.3897/zookeys.762.23926

**Published:** 2018-05-30

**Authors:** Yuanhao Ren, Zhaohui Li, Feng Zhang

**Affiliations:** 1 Institute of Applied Ecology, Nanjing Xiaozhuang University, Nanjing 211171, P. R. China; 2 Department of Entomology, College of Plant Protection, Nanjing Agricultural University, Nanjing 210095, P. R. China

**Keywords:** chaetotaxy, Dicranocentrus hainanicus sp. n., Heteromurinae

## Abstract

A new species, *Dicranocentrus hainanicus* Ren & Zhang, **sp. n.**, is described from Hainan Province, China. Complete tergal chaetotaxy including microchaetae is illustrated and discussed. It is characterized by having the dental spines arranged in 2–3 rows, two inner teeth on unguis, 5, 2, 2 central macrochaetae on Abd. I–III, two inner S-chaetae on Abd. V displaced anteriorly, and the additional microchaetae associated with the S-chaeta acc.p6 on Th. II–Abd. II. It is most similar to *D. chenae* Ma, Chen & Soto-Adames but differs from the latter in the number and arrangement of dental spines and the absence of macrochaeta Pa1 on dorsal head. A key to the Chinese species of the genus is provided.

## Introduction

The genus *Dicranocentrus* Schött, 1893 is widespread in pantropical regions. It is characterized by having the first and the second antennal segments subdivided, the third and the fourth antennal segments annulated, eyes 8+8, prelabral chaetae not bifurcated, postantennal organ absent, dental lobe without compound spines, dental spines present or absent, and the mucro bidentate with a basal spine (Cipola et al. 2016). Mari-Mutt (1979) made great contribution to its biology, character assessments, and phylogeny, and further divided it into three groups based on dorsal cephalic chaetotaxy. Posterior macrochaetae on dorsal head are absent in *marias*-group, and macrochaeta S2 is present in *sundanensis*-group but absent in *gracilis*-group. *Dicranocentrus* belongs to Heteromurini (Heteromurinae) due to the presence of body scales (Zhang and Deharveng 2015) but its relationships to other genera of Heteromurini are still unclear (Mari-Mutt 1980; Cipola et al. 2016). Dorsal cephalic and tergal macrochaetotaxy, differentiated chaetae on tibiotarsi, tenent hairs, dental spines are the main diagnostic characters widely used by Mari-Mutt and subsequent authors. Soto-Adames and Anderson (2017) attempted to explore the complete idiochaetotaxy (including macrochaetae, microchaetae, partial S-chaetae) for the first time in *Dicranocentrus*; homology of some elements were uncertain due to the absence of chaetotaxic modification during postembryonic development. These characteristics are usually unknown in four species from China: *D. indicus* Bonet, 1930 from Taiwan, *D. chenae* Ma, Chen & Soto-Adames, 2006 from Guangxi, *D. wangi* Ma & Chen, 2007 from Guangdong and *D. liuae* Xu & Zhang, 2014 from Anhui. Here a new species is described from the southernmost province of China, Hainan.

## Materials and methods

Specimens were cleared in Nesbitt’s fluid, mounted under a coverslip in Hoyer’s solution, and studied using a ZEISS AXIO Scope.A1 microscope and Axiocam camera. Dorsal body chaetae nomenclature follows Szeptycki (1979), dorsal cephalic chaetae follow Mari-Mutt (1979) and Soto-Adames (2008), inter-ocular chaetae follow Mari-Mutt (1986), tergal S-chaetae follow Zhang and Deharveng (2015), labial palp follows Fjellberg (1999), clypeal chaetae follow Yoshii and Suhardjono (1992) and Zhang et al. (2016), and labial chaetae follow Gisin (1967). The number of macrochaetae is given by half-tergite in the descriptions. Symbols representing chaetal elements used in the figures are as follows: circle, chaeta; cross, bothriotrichum; circle with a slash, pseudopore; “v”, scales. All materials are deposited in the collections of the Department of Entomology, College of Plant Protection, Nanjing Agricultural University (NJAU), P. R. China.

### Abbreviations

**Th.** thoracic segment;

**Abd.** abdominal segment;

**Ant.** antennal segment;

**mac** macrochaeta/ae;

**mes** mesochaeta/ae;

**mic** microchaeta/ae;

**ms** S-microchaeta/ae;

**sens** ordinary tergal S-chaeta/ae.

## Taxonomy

### Dicranocentrus hainanicus

Taxon classificationAnimaliaCollembolaEntomobryidae

Ren & Zhang sp. n.

http://zoobank.org/85C9388D-C78D-4D2E-8E3B-6A4C8AA50375

Figs 1–8 , 9–14 , 15–26 , 27–28

#### Material.

Holotype: ♂ on slide, China, Hainan Province, Wuzhi Mountain, 18.903°N, 109.688°E, altitude ca. 901 m, 29 Dec 2015, DY Yu leg. (#15HN5). Paratypes: four ♀♀ on slides and three juveniles and one adult in alcohol, same data as holotype. All deposited in NJAU.

#### Etymology.

Named after the type locality where the new species was collected.

#### Diagnosis.

No obvious color pattern. Mac S2 present and mac Pa1 absent on dorsal head. Labial chaetae l_1_ and l_2_ smooth. Inner tibiotarsi and manubrium dorsally with smooth chaetae. Unguis with two inner teeth and without unpaired tooth. Tenent hairs acuminate. Den with 30–41 inner spines arranged in 2–3 rows. Th. II with two medio-medial, two medio-sublateral and eight posterior mac, and mac p5 present. Abd. I–IV with 5, 2, 2, 5 central mac. An additional mic associated with acc.p6 present on Th. II–Abd. II. Tergal sens as 2, 2|1, 3, 3, (3+≈35), 4; on Abd. V two inner sens anterior to lateral two.

#### Description.

**Figures 1–8.**
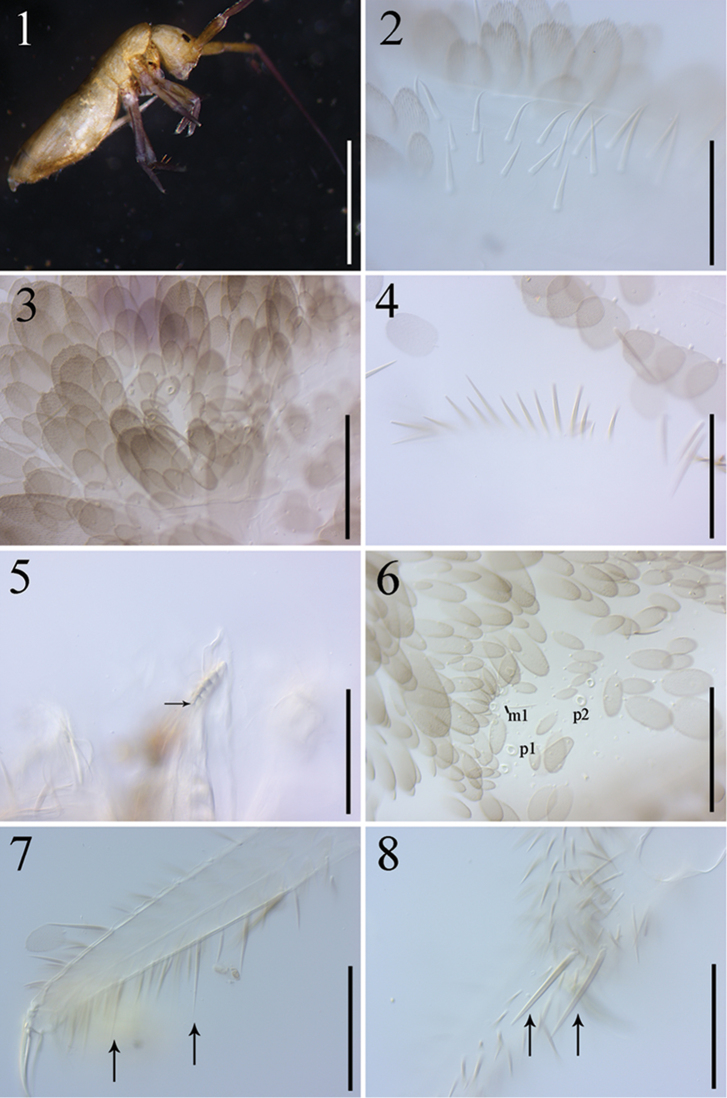
*Dicranocentrus hainanicus* sp. n. **1** habitus **2** left Ant. Ia (dorsal side) **3** dorsal head **4** distal chaetae along posterior margin of head **5** right mandible **6** Th. III **7** tibiotarsus I **8** external side of tibiotarsus II. Scale bars: 1.5 mm (**1**); 50 µm (**2**); 125 µm (**3–8**).

Body length (head + thorax + abdomen) up to 3.96 mm. Ground color yellow. Antennae gradually dark purple towards tip. Eye patches dark (Fig. 1). Scales rounded, truncate, or pointed with numerous short striations; scales present on Ant. I–II, body, legs, both sides of head, ventral tube and manubrium, and ventral side of dens (Figs 2–4, 6, 8, 10). Scales in the posterior row along tergal margin much larger than anterior ones. Scales on dens much narrower.

**Figures 9–14.**
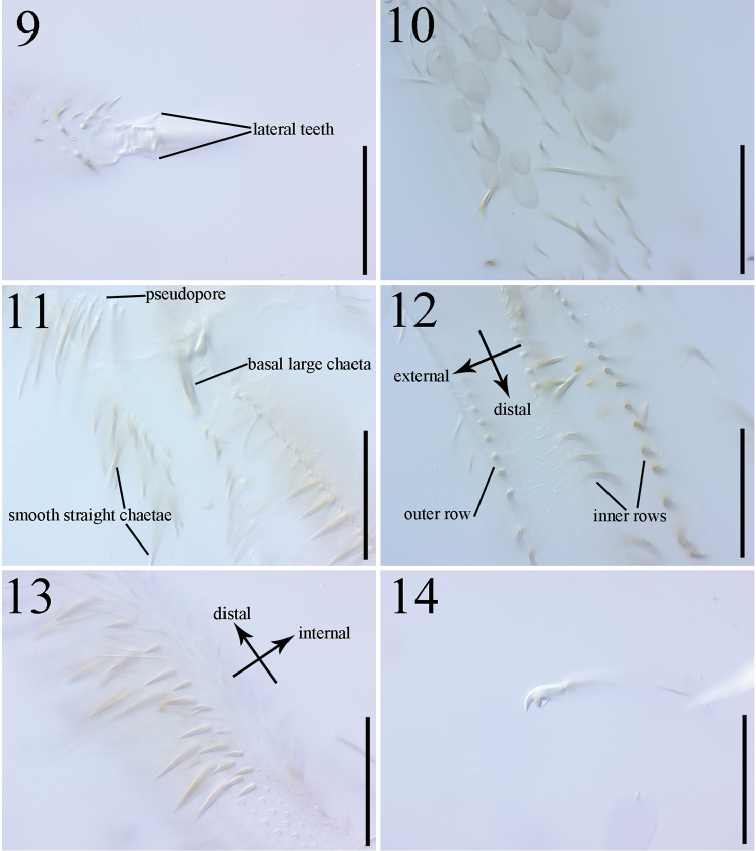
*Dicranocentrus hainanicus* sp. n. **9** unguis II external view **10** dorsal side of manubrium **11** manubrial plaque and base of dens dorsal view **12** dens dorsal view **13** dental spines **14** mucro. Scale bar: 125 µm.

**Figures 15–26.**
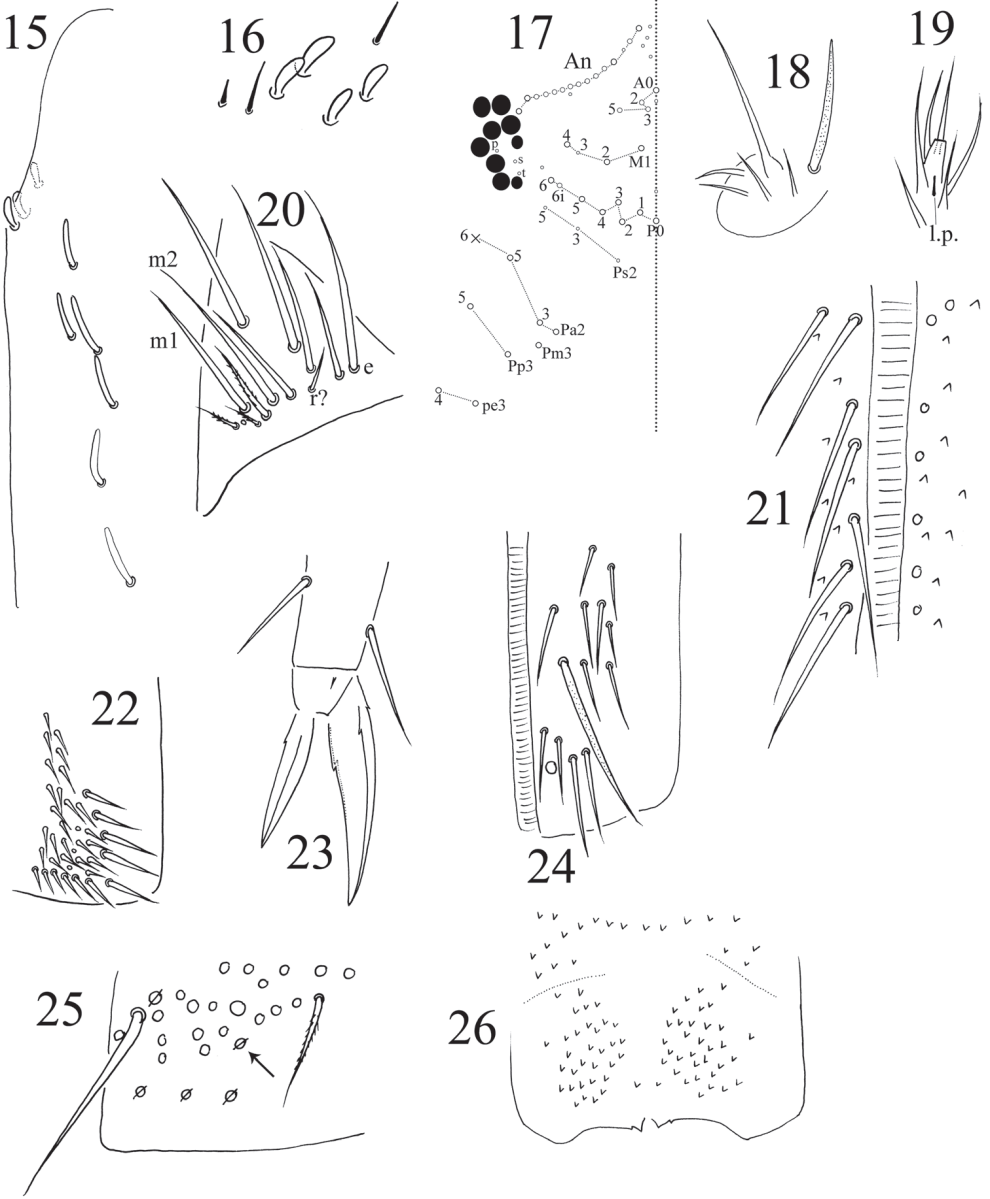
*Dicranocentrus hainanicus* sp. n. **15** Ant. I organ **16** left Ant. III organ **17** dorsal cephalic chaetotaxy **18** maxillary outer lobe (right side) **19** lateral process of labial palp (left side) **20** labial triangle (right side) **21** postlabial chaetae along groove **22** trochanteral organ **23** hind claw (posterior side) **24** anterior face of ventral tube **25** manubrial plaque (right side), arrow indicating that it is often absent **26** distal part of manubrium ventrally.

Antenna approx. 3.5 times as long as cephalic diagonal. Antennal segment ratio as Ia: Ib: IIa: IIb: III: IV= 1: 4.04–5.29: 1.19–1.57: 4.57–6.29: 13.04–18.08: 4.71; ratio in holotype as 1: 4.04: 1.26: 4.57: 13.04 (Ant. IV lost). Ant. III distally and IV annulated and often fused. Smooth spiny mic at base of antennae indistinctly separated from ordinary mic and thus their number unclear (Fig. 2). Swollen S-chaetae of antennal organ apico-laterally 3–4, 3, 4 on Ant. Ib, IIb and III, respectively. Subcylindrical, thick S-chaetae 6–7, 3–4, 7 ventro-laterally on Ant Ib, IIb and III, respectively (Figs 15, 16). Pseudopores 2, 2, 2 ventro-apically on Ant. I, II, III, respectively. Ant. IV without apical bulb but its apex with a pin chaeta.

Eyes 8+8. Labral papillae four, all with a pointed tip and outer two slightly larger. Prelabral and labral chaetae 4/5, 5, 4, all smooth. Clypeal chaetae ciliate on prefrontal and frontal areas but their number unclear; lateral L_1_ and L_2_ smooth. Dorsal cephalic chaetotaxy with 13–16 antennal (An), four anterior (A), three median (M), eight sutural (S), one postocular (Po=Pa5) mac, and seven (Pa2–3, Pm3, Pp3, Pp5, Pe3–4) posterior (P) mac; mac Pa1 absent; inter-ocular chaetae as p, s, t (Fig. 17). A transverse of small ciliate chaetae present along posterior margin (Fig. 4). Mandibles with 4+6 apical teeth; five apical teeth much larger than distal one on right mandible (Fig. 5). Maxillary outer lobe with four smooth sublobal hairs (Fig. 18). Labial papillae A–E with 0, 5, 0, 4, 5 guard chaetae, respectively; lateral process of papilla E thin, with tip not reaching apex of labial papilla (Fig. 19). Labium with five smooth proximal and five smooth anterior (a1–5) chaetae; submentum chaetae 8–13, 0–3 of them ciliate (Fig. 20), chaetae l_1_ and l_2_ smooth. Postlabial chaetae 6–7 smooth chaetae along cephalic groove (Fig. 21).

Trochanteral organ with 29–44 smooth spine-like chaetae; 12–13 in L-shaped arms and 17–31 between arms (Fig. 22). Tibiotarsi with smooth inner chaetae (Fig. 7) and few large, ciliate outer chaetae, but their number unclear because partial chaetae lost with only large sockets seen (Fig. 8). Unguis with two inner and two lateral teeth; unpaired inner and outer teeth not seen. Unguiculus lanceolate with a tiny outer tooth. Tenent hairs acuminate (Figs 9, 23). Abd. IV 1.24–1.53 times as long as Abd. III along dorsal midline. Ventral tube anteriorly with 14–17 weakly ciliate chaetae, two of them much larger (Fig. 24); posteriorly with more than 50 chaetae; each lateral flap with more than 40 chaetae and some of them ciliate. Tenaculum with 4+4 teeth, corpus with approx. six ciliate chaetae. Manubrium dorsally with 1+1 lateral rows of smooth straight chaetae, which are also present manubrial plaque (Fig. 25) and dental base (Fig. 11). Manubrial plaque with 4–5 pseudopores and 14–17 ciliate chaetae on each side (Fig. 25). Distal manubrium ventrally with 33–41 scales (Fig. 26). Dens dorsally with two rows of ciliate chaetae; inner ones stronger than outer ones (Fig. 12); the most basal chaeta of inner row extremely thicker and longer (Fig. 11). Den internally with 30–41 (33 in holotype) basal spines arranged roughly in 2–3 rows; basal ones more dense than distal ones; outer spines larger than inner ones (Fig. 13). Smooth distal part of dens 4.70–6.25 times as long as mucro; apical tooth slightly longer than subapical one; mucronal spine just reaching the apex of subapical tooth (Fig. 14).

**Figures 27–28.**
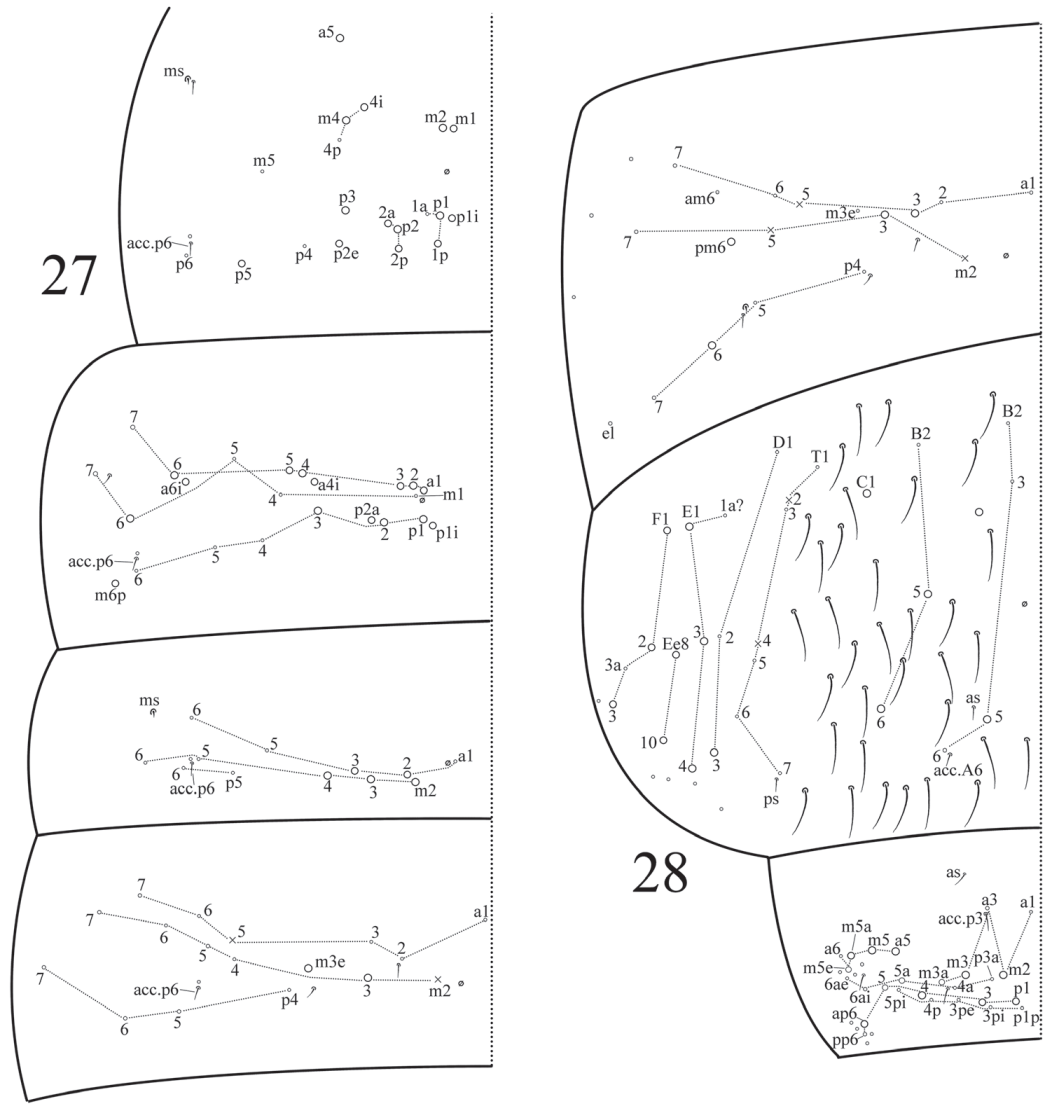
*Dicranocentrus hainanicus* sp. n. Tergal chaetotaxy. **27** Th. II–Abd. II **28** Abd. III–V.

Th. II with two (m1, m2) medio-medial, two (m4, m4i) medio-sublateral and eight (p1, p1i, p1p, p2, p2a, p2p, p2e, p3, p5) posterior mac; m4p, m5, p1a, p4, p6 as mic. Th. III with 15 (a1–6, a4i, a6i, m6, p1–3, p1i, p2a, m6p) mac; a7 and m7 as mes; m1, m4–5 and p4–6 as mic. Abd. I with five (a2–3, m2–4) mac; a1, a5–6, m5–6, p5–6 as mic. Abd. II with two (m3, m3e) mac; a2–3, a5–6, m4–7 and p4–7 as mic (Fig. 27). Abd. III with two (a3, m3) central and two (pm6, p6) lateral mac; a1–2, a6–7, am6, m7, p4–5, and p7 as mic; m4 absent. Abd. IV with five (A5, B5–6, C1, a mac of unclear homology) central and nine (D3, E1, E3–4, F1–3, Ee8, Ee10) lateral mac. Abd. V with 13 (m2–3, m3a, a5, m5, m5a, m5e, p1, p3–5, ap6, p5a) mac (Fig. 28). Abd. VI without smooth chaetae. Tergal ms as 1, 0|1, 0, 1, 0, 0. Sens as 2, 2|1, 3, 3, (3+≈35), 4; Abd. IV with three ordinary sens (as, ps, acc.A6) and approx. 35 elongated sens; inner two sens anterior to lateral two on Abd. V. An additional mic associated with acc.p6 and of unclear homology present on Th. II–Abd. II.

#### Ecology.

In litter of coniferous and broad-leaved mixed forests.

#### Remarks.

As a member of *sundanensis*-group (cephalic mac S2 present), *D. hainanicus* sp. n. is similar to *Dicranocentrus chenae* Ma, Chen & Soto-Adames in most features including two inner teeth on unguis and tergal macrochaetae, but differs from it in the absence of mac Pa1 on dorsal head, mac p5 present on Th. II and the abundant (30–41) dental spines arranged in 2–3 rows (single row in *D. chenae*). The absence of outer tooth on unguis is also interesting but the detail is often lacking or overlooked in descriptions of known species.

Nearly complete dorsal chaetotaxy is again illustrated and compared with Soto-Adames and Anderson (2017). On dorsal head, a mic between the outer two mac of series M is homologous to m3. For the two mac external to S5, the outer mac, having a larger socket than inner one, is supposed to be the primary chaeta S6. On Th. II, the most internal four posterior chaetae labeled as “p1, p2, p2a, p2p” in *D. icelosmarias* are named here as p1i, p1, p1a, p1p in the light of the presence of the set p2+ (p2, p2a, p2p) between p1 and p3. On Th. III, “p1, p2” in are possibly homologous to p2 and p2a in *D. hainanicus* sp. n. Mic p5 on Abd. I and mic m4 on Abd. II are absent in *D. icelosmarias* and *Heteromurus nitidus* but present in new species. On Abd. III, mic m4 is present in *D. icelosmarias* and *H. nitidus* but absent in *D. hainanicus* sp. n. On Abd. IV homologies of many chaetae are difficult to determine in the absence of sufficient evidence across Entomobryoidea; three ordinary sens are observed as those on Abd. II–III and named as as, acc.A6 and ps, respectively. On Abd. V, the number of sens is identical to those reported in Zhang and Deharveng (2015), therefore the inner two are supposed to as and acc.p3 here although their positions displace more anteriorly compared to *D. wangi* and *D. liuae*.

##### Key to the species of *Dicranocentrus* from China

**Table:** 

1	Abd. I with 5+5 mac (Fig. 27)	**2**
–	Abd. I with 3+3 mac	**3**
2	Mac Pa1 present on dorsal head; Th. II without p5 mac; dens with 15–19 inner spines arranged in a row	***chenae* Ma, Chen & Soto-Adames, 2006**
–	Mac Pa1 absent on dorsal head (Fig. 17); Th. II with p5 mac; dens with 30–41 inner spines arranged 2–3 rows	***hainanicus* sp. n.**
3	Abd. III with 1+1 central mac	***liuae* Xu & Zhang, 2014**
–	Abd. III with 2+2 central mac (Fig. 28)	4
4	Abd. II with 3+3 mac; Abd. III with 3+3 lateral mac	***indicus* Bonet, 1930**
–	Abd. II with 2+2 mac (Fig. 27); Abd. III with 2+2 lateral mac (Fig. 28)	***wangi* Ma & Chen, 2007**

## Supplementary Material

XML Treatment for Dicranocentrus hainanicus
